# Effects of Oral Vitamin C Supplementation on Liver Health and Associated Parameters in Patients With Non-Alcoholic Fatty Liver Disease: A Randomized Clinical Trial

**DOI:** 10.3389/fnut.2021.745609

**Published:** 2021-09-14

**Authors:** Zhangya He, Xiaomin Li, Hexiang Yang, Pei Wu, Shanshan Wang, Dan Cao, Xiaoxiao Guo, Zhangrui Xu, Jiayi Gao, Wanyu Zhang, Xiaoqin Luo

**Affiliations:** ^1^Department of Nutrition and Food Safety of School of Public Health, Xi'an Jiaotong University, Xi'an, China; ^2^Department of Clinical Nutrition, Xianyang Central Hospital, Xianyang, China; ^3^Emergency Medical Center, Xi'an Public Health Center, Xi'an, China; ^4^Department of Shaanxi Health Supervision Center, Xi'an, China

**Keywords:** vitamin C, NAFLD, glucose metabolism, adiponectin, microbiota

## Abstract

Non-alcoholic fatty liver disease (NAFLD) is now recognized as the most prevalent hepatic disorder worldwide, and an unhealthy lifestyle is the leading risk factor for its occurrence. Vitamin C (VC) has been suggested to protect NAFLD, whereas evidence from randomized controlled trials (RCTs) is sparse. In this study, we aimed to investigate the potential benefits of VC supplementation daily on liver health and associated parameters in patients with NAFLD. In this double-blind, RCT, 84 patients with NAFLD, aged 18–60 years old, were assigned to 12 weeks of oral treatment with either low (250 mg/day, *n* = 26), medium (1,000 mg/day, *n* = 30), or high (2,000 mg/day, *n* = 28) doses of VC supplements. After the intervention, the Medium group had a more significant decrease in aspartate aminotransferase [Medium, −5.00 (−10.25, −1.75) vs. High, −2.50 (−7.75, 0.00), *P* = 0.02] and alanine aminotransferase [Medium, −8.00 (−18.00, −1.75) vs. High, −3.50 (−13.75, 4.25), *P* = 0.05; Medium vs. Low, −3.00 (−9.00, 5.50), *P* = 0.031]. The levels of other indicators of liver health, such as gamma-glutamyl transferase, alkaline phosphatase, total bilirubin, and direct bilirubin were decreased after the intervention but comparable among the three groups and so did the parameters of glucose metabolism, such as fasting insulin, fasting glucose, and homeostasis model assessment for insulin resistance. The plasma level of VC in patients and total adiponectin and high molecular weight (HMW) adiponectin levels were also elevated but not in a dose-dependent manner. Meanwhile, analysis of fecal microbiota composition showed an increase in the alpha diversity (Abundance-based Coverage Estimator (ACE), Shannon, chao1, and Simpson) both in the Low and the Medium groups. A total of 12 weeks of VC supplementation, especially 1,000 mg/day, improved liver health and glucose metabolism in patients with NAFLD. The elevated plasma levels of VC, total and HMW adiponectin, and the improvement of intestinal microbiota may have made some contributions.

## Introduction

Non-alcoholic fatty liver disease (NAFLD) is recognized as the most common chronic liver disease globally, with almost 25% prevalence in the general population (1). In the early 2000s, the prevalence of NAFLD in China was 23.8% and reached 32.9% in 2018, which was responsible for over a third (37%) of the incidence of central obesity or diabetes (2, 3). Without intervention, NAFLD may progress from simple hepatic steatosis to advanced steatohepatitis, even to fibrosis, cirrhosis, and hepatocellular carcinoma (4). Therefore, identifying approaches to prevent NAFLD is one of the public health problems that need to be solved urgently.

Previous studies have reported the impact of oxidative stress and inflammation on NAFLD (5–7). Vitamin C (VC), as an effective water-soluble antioxidant, has a scavenging effect on excessive free radicals in the body and a protective effect on tissue damage caused by oxidative stress, and it probably plays a protective role against NAFLD (8, 9). The association between dietary VC intake and NAFLD has been studied, but not extensively, and with conflicting results (10–13). Han et al. revealed a significant positive association between low VC intake and NAFLD in the male population in a cross-sectional study (11). In contrast, another small sample cross-sectional study suggested that both dietary VC intake and plasma VC concentration were of similar levels between patients with NAFLD and healthy controls (12). Moreover, the clinical burden of NAFLD is not only confined to liver-related morbidity and mortality, but NAFLD is a multisystem disease affecting extrahepatic organs and regulatory pathways (14). Epidemiological studies have also shown an association between VC status and reduced insulin resistance and improved blood glucose control (15–17). Meanwhile, higher plasma VC was associated with a lower risk of developing type 2 diabetes (T2DM) (18). A previous study suggested that supplementation of the diet with kiwifruit, which is rich in VC, enhanced plasma VC status and also decreased the HbA1c levels (19). A recent meta-analysis investigating VC supplementation and insulin resistance found that doses of ≥200 mg/day VC significantly reduced glucose concentrations in patients with T2DM, mainly if the intervention was for more than 30 days and in older individuals (20).

However, the possible mechanisms of VC in improving liver health and glucose metabolism remain unclear. Adiponectin, which exerts marked insulin-sensitizing and anti-inflammatory effects (21), has been reported to be involved in the pathogenesis of non-alcoholic fatty liver, and a previous study demonstrated that a decrease in adiponectin levels is an independent risk factor of developing NAFLD (22, 23). Interestingly, our previous study and other study showed that VC treatment could increase the secretion of high molecular weight (HMW) adiponectin from human hepatocytes and adipocytes (24, 25). In addition to having lower levels of adiponectin, patients with NAFLD also have increased intestinal permeability and the changes in intestinal microbiota may also be the driving force for the progress of NAFLD (26–29). However, the effect of VC on the intestinal microbiota is unclear.

By now, no studies have been performed to determine whether VC alone has a causal effect on liver function and glucose homeostasis parameters in patients with NAFLD. Therefore, in this double-blind, randomized controlled trial (RCT), we aimed to find out whether VC supplementation could improve liver function and other associated metabolic markers in NAFLD individuals and explored possible mechanisms.

## Patients and Methods

### Patients

From March to May 2020, patients with NAFLD aged 18–60 years old and who have lived in Xianyang for more than 10 years were recruited from the Medical Examination Center of Xianyang Central Hospital, Xi'an, Shaanxi, China. Patients who were newly diagnosed with NAFLD were included (30). Patients affected by hepatitis B and C, and those with cardiac, renal, hyperuricemia, hyperoxaluria, hemochromatosis, autoimmune, cirrhosis, and other metabolic diseases were excluded. Exclusion criteria also included insulin treatment, smoking habits, alcohol intake (>20 g/day), other antioxidant supplements or medications taking in the past 3 months, recreational or statins drug use, and exposure to environmental toxins known to induce liver steatosis. According to the sample size calculation formula, the number of cases required for each group was 25. Taking a dropout rate of 20% into consideration, at least 90 volunteers will be recruited. Overall, 98 participants were enrolled in the study. This trial was registered at ClinicalTrials.gov (http://www.chictr.org.cn); Identifier: ChiCTR2000033171. The study was consistent with the ethical guidelines of the Helsinki 1975 Declaration and was approved by the Ethics Committee of Xi'an Jiaotong University and Xianyang Central Hospital. Signed informed consents were obtained from all the participants, and all of them were told that there is no need to change eating habits except for the intervention.

### Study Supplements and Allocation

Participants were assigned to one of three different treatment groups using stratified randomization. Randomization was stratified by gender, age, weight, body mass index (BMI), and waist circumference. Based on the Chinese Residents' Dietary Guidelines, the proposed intake for chronic non-communicable diseases (PI-NCD) is 200 mg/day for adults (31). Since it was tough to produce a placebo with the same taste as VC (Blackmores, Australia), the Low (dose) group took one tablet of VC (250 mg/day) per day, and the High group was supplemented with 2,000 mg/day, which is the tolerable upper intake level. The Medium group was also designed and assigned to 1,000 mg/day of VC. Participants were asked to take or chew the supplements before meals. During the 12 weeks intervention time, they were also asked to visit the intervention staff every 2 weeks where their clinical symptoms and side effects from VC supplementation were assessed, compliance was checked based on the number of unconsumed tablets, and tablets for further use were dispensed. In addition, subjects were instructed to maintain the same lifestyle and diet during the trial as usual.

### Procedures

Participants were asked to provide demographic information and completed self-administered diet and physical activity questionnaires before the intervention. Anthropometric parameters (body weight, height, and waist and hip circumferences) and systolic and diastolic blood pressures were assessed according to standard methods. BMI was calculated as body weight divided by height squared (kg/m^2^). Fasting blood was collected at baseline and week 12 by an antecubital venous puncture. Serum or plasma samples were centrifuged at 1,500 g for 15 min at 4°C and frozen at −80°C. Plasma VC content was detected by 1,290 Infinity Ultrahigh-Performance Liquid Chromatography (UHPLC) from Agilent Company in Guangzhou KingMed Center for Clinical Laboratory Co., Ltd., Guangdong Province, China. The detection method has been described in detail previously (32). Briefly, the samples were pretreated by protein precipitation, centrifugation, etc. Then, the processed samples were introduced into the UHPLC system with the mobile phase and then chromatographically separated to reach the diode array detector. Finally, the VC concentration was calculated by the strength of the signal response. All other blood indicators associated with the liver function such as total protein (TP), aspartate aminotransferase (AST), alanine aminotransferase (ALT), gamma-glutamyl transferase (γ-GT), alkaline phosphatase (ALP), total bilirubin (TBIL), serum indirect bilirubin (IBIL), serum direct bilirubin (DBIL), total cholesterol (TC), and triglyceride (TG) were tested twice in parallel using Hitachi 7600 automatic biochemical analyzer (Hitachi, Tokyo, Japan) to obtain the average value. Plasma human total and HMW adiponectin were measured using ELISA test kits purchased from Abcam in the United Kingdom (Cat. # ab99968) and MERCK, Germany (Cat. # EZHMWAN-65K), respectively. Finally, the absorbance (Optical Delnsity, OD value) was measured at a wavelength of 450 nm with a microplate reader to calculate the concentration.

We also collected the feces of volunteers from the Low and Medium groups at baseline and week 12 for 16S rDNA Amplicon Sequencing (Novogene Company, China). Operational taxonomic units (OTUs) clustering and species classification analysis based on the clean data obtained by sequencing were performed. According to the OTU clustering results, species annotations were made on the representative sequence of each OTU, and the corresponding species information and species-based abundance distribution were obtained. At the same time, the abundance, alpha diversity calculation, Venn map, and petal map analysis of OTUs were performed to obtain the species richness and uniformity information among different samples or groups.

### Statistical Analysis

Differences in baseline measurements among groups were assessed using the chi-squared test for categorical variables and variance tests for continuous measures. The one-way ANOVA test and the Student–Newman–Keuls test were used to determine the significance of the difference. In non-parametric analyses, the significance of the changes within and between groups was assessed by the Wilcoxon rank-sum test. Analyses were carried out using SPSS software (version 24) (SPSS Inc., Chicago, USA). Significance was defined as *P* < 0.05 (two-tailed).

## Results

### Baseline Characteristics

**Figure 1:**
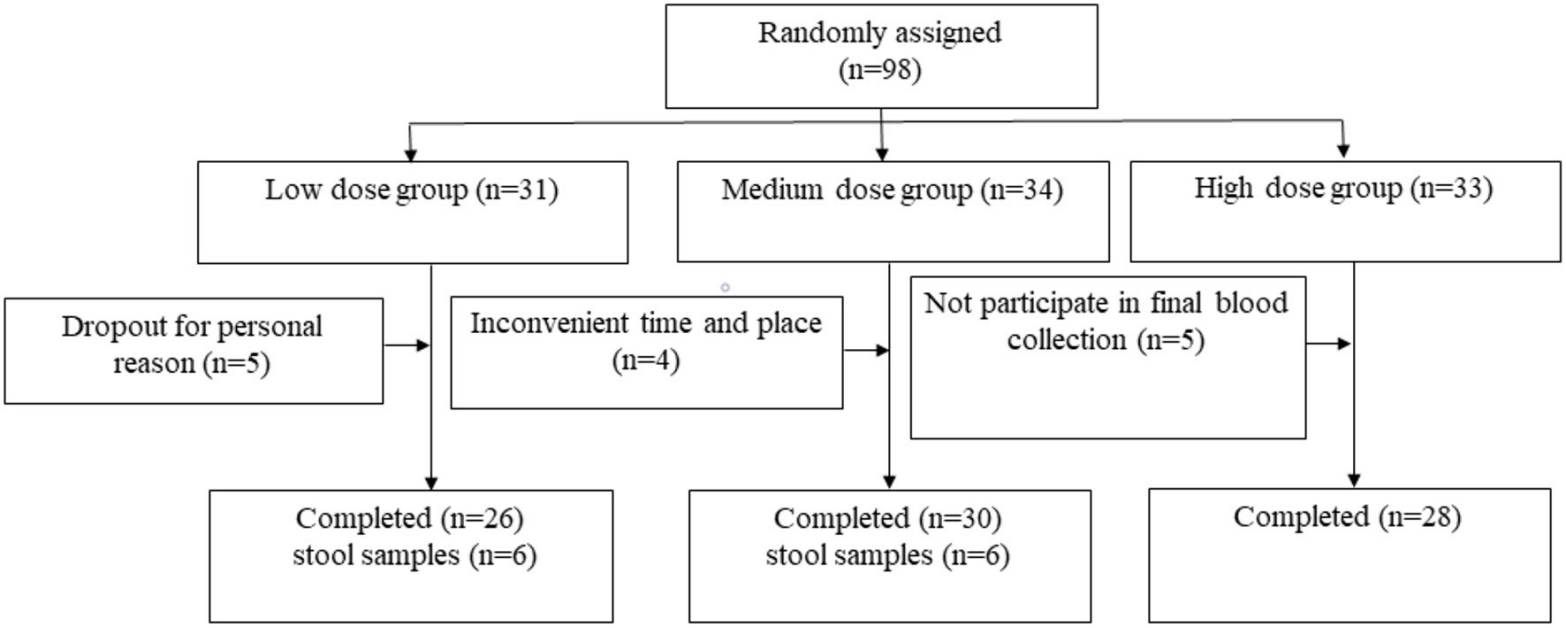
Flow diagram of participant progress through the study. ^1^means nine participants provided samples before the intervention, three participants lose after the intervention, six participants from the Low group provided samples both before and after the intervention finally. ^2^means seven participants provided samples before the intervention, one participant lose after the intervention, six from the Medium group provided samples both before and after the intervention finally. ^3^means five participants provided samples before the intervention, all participants lose after the intervention, there are no complete samples finally in the High group.

**Figure 2:**
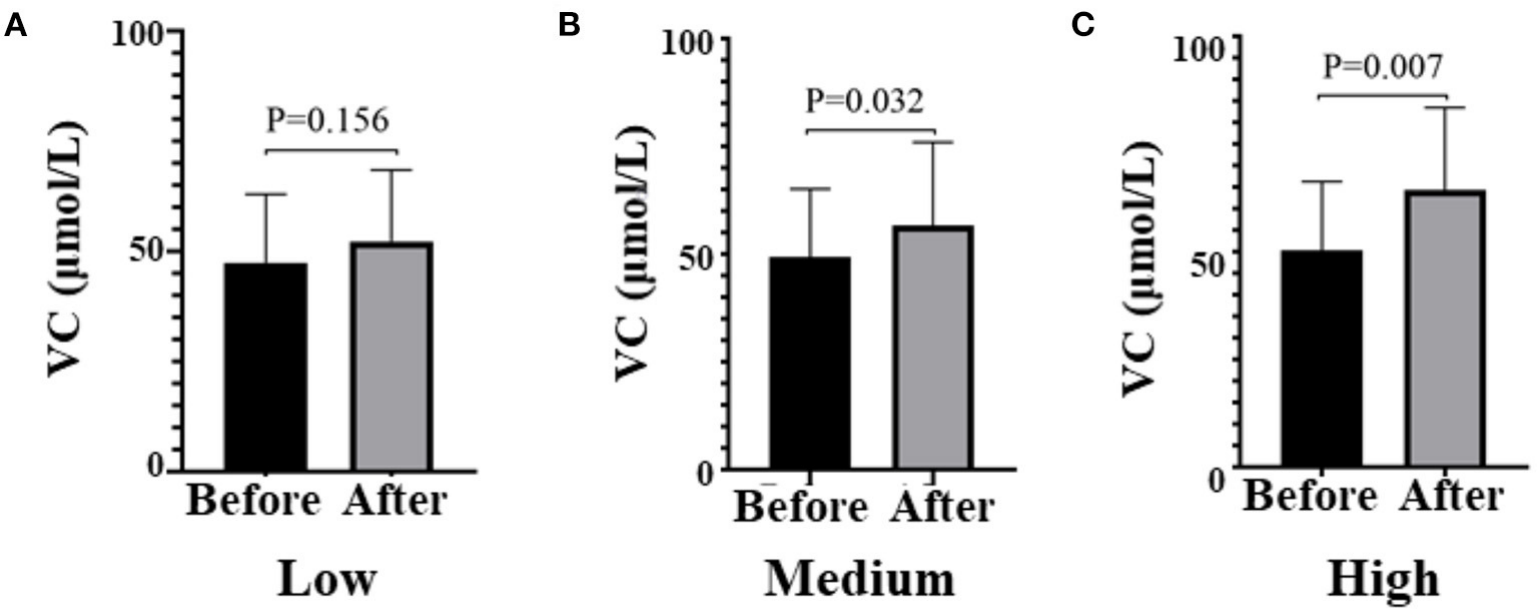
Plasma VC levels before and after the intervention in all three groups, respectively. **(A)** means Low dose group, **(B)** means Medium dose group, **(C)** means High dose group. Black means VC plasma concentration (μmol/l) before the intervention, gray means VC plasma concentration after the intervention; paired *t*-test used to test the difference before and after the intervention in three groups. VC, vitamin C.

Of 98 subjects enrolled, 84 completed the study (Figure 1). A total of 14 subjects terminated early. Five withdrew due to personal reasons, four lost due to inconvenience, and five were lost to follow-up. The samples of the stool of volunteers were collected before and after the intervention. Only 12 people, 6 from the Low group and 6 from the Medium group, provided samples both before and after the intervention. The mean age of participants was 40.6 years, and 58.3% were women. The fasting glucose, lipids, and plasma VC status were comparable among the three groups at baseline (Table 1). After the intervention, plasma VC levels were significantly elevated in the Medium group (before 56.67 ± 19.27 vs. after 49.28 ± 15.82, *P* = 0.032) and the High group (before 64.40 ± 19.06 vs. after 50.27 ± 16.03, *P* = 0.007; Figure 2). Interestingly, men had lower plasma VC levels before the intervention than women (men 39.90 ± 13.73 vs. women 55.55 ± 13.64, *P* < 0.001). Nevertheless, after the intervention, there was a remarkable increase in the men among the three groups (*P* = 0.024). There was also an increase of plasma VC levels in the women before and after the intervention, but without static significance (*P* = 0.018; Supplementary Figure 1).

**Table 1:** Baseline characteristics of participants^a^.

**Characteristics**	**Total (*n* = 84)**	**Low (*n* = 26)**	**Medium (*n* = 30)**	**High (*n* = 28)**	***P*** **-value^b^**
Women	49 (58.3)	16 (61.5)	17 (56.7)	16 (57.1)	0.92
Age, y	40.60 ± 7.82	39.96 ± 7.76	42.07 ± 7.81	39.59 ± 7.94	0.44
Weight, kg	70.39 ± 9.36	70.21 ± 9.04	69.76 ± 9.28	71.21 ± 9.99	0.84
BMI, kg/m^2^	25.46 ± 2.45	25.36 ± 2.16	25.37 ± 2.41	25.66 ± 2.80	0.87
Waist circumference, cm	90.11 ± 8.27	89.96 ± 7.55	90.98 ± 8.47	89.33 ± 8.88	0.75
Glucose, mmol/l	5.10 (0.64)	5.14 (0.80)	5.00 (0.76)	5.20 (0.55)	0.29
TC, mmol/l	4.60 (0.70)	4.79 (1.14)	4.64 (0.39)	4.39 (0.67)	0.07
TG, mmol/l	1.62 (1.20)	1.84 (1.20)	1.62 (1.35)	1.23 (1.22)	0.58
VC, μmol/l	49.03 ± 15.65	47.40 ± 15.52	49.28 ± 15.82	50.27 ± 16.03	0.80

a *Values are means ± SDs, median (interquartile range), and n (%) for variables with normal distribution, variables with skewed distribution, and categorical variables, respectively. BMI, body mass index; TC, total cholesterol; TG, triglycerides; VC, vitamin C.*

b *Among-group differences were compared using the ANOVA test and the χ^2^ test for continuous variables and categorical variables, respectively*.

### Positive Modulatory Effect on Liver Function

As shown in Table 2, after the intervention, some serological indicators of the three groups all improved statistically. First, in the low group, compared with baseline, the AST, ALP, TBIL, and DBIL indexes of the Low group decreased. In the Medium group, AST, ALT, γ-GT, ALP, and DBIL indexes decreased. In the High group, the levels of γ-GT, ALP, TBIL, DBIL, and triglycerides also decreased. Second, compared among groups, the changes of all the liver function indicators were similar except for ALT and AST, whose *P*-values were close to 0.05. Further comparison of specific differences between groups showed that compared with the High group, the Medium group had a more significant decrease in AST [Medium, −5.00 (−10.25, −1.75) vs. High, −2.50 (−7.75, 0.00), *P* = 0.02]. Medium group had a more significant decrease in ALT [Medium, −8.00 (−18.00, −1.75) vs. High, −3.50 (−13.75, 4.25), *P* = 0.05; Medium, −8.00 (−18.00, −1.75) vs. Low, −3.00 (−9.00, 5.50), *P* = 0.031]. However, there was no statistically significant difference in ALT or AST between the Low and the High groups.

**Table 2:** Metabolic indicators change during the trial^a^.

**Characteristic**		**Low (*n* = 26)^b^**	**Medium (*n* = 30)^c^**	**High (*n* = 28)^d^**	***P*** ^group^	***P*** ^1v2^	***P*** ^2v3^	***P*** ^1v3^
TP, g/l	Before	73.75 (70.48, 76.43)	74.90 (72.00, 76.43)	73.10 (71.73, 76.53)				
	After	72.75 (70.43, 77.20)	73.80 (71.00, 77.23)	74.55 (72.50, 76.55)				
	Change	−0.20 (−2.75, 1.33)	−1.30 (−2.75, 1.55)	0.70 (−3.55, 4.45)	0.601	–	–	–
Albumin, g/l	Before	45.45 (44.68, 48.25)	46.10 (44.05, 48.25)	46.45 (44.63, 47.70)				
	After	46.10 (44.88, 47.20)	46.45 (44.00, 47.45)	46.55 (45.30, 48.30)				
	Change	0.1 (−2.08, 1.93)	0.25 (−0.95, 1.875)	0.35 (−0.65, 1.93)	0.823	–	–	–
AST, U/l	Before	23.00 (19.75, 26.00)	23.00 (18.00, 26.00)	23.00 (17.25, 29.75)				
	After	17.50 (16.00, 22.00)	18.50 (15.75, 21.25)	18.50 (15.25, 24.75)				
	Change	−3.50 (−9.50, 2.00)^*^	−5.00 (−10.25, −1.75)^**^	−2.50 (−7.75, 0.00)	0.053	0.086	0.020	0.565
ALT, U/l	Before	25.00 (16.75, 33.25)	26.00 (19.75, 33.25)	28.50 (20.00, 45.75)				
	After	19.00 (13.00, 31.25)	19.50 (12.75, 31.75)	20.00 (14.50, 43.50)				
	Change	−3.00 (−9.00, 5.50)	−8.00 (−18.00, −1.75)^*^	−3.50 (−13.75, 4.25)	0.056	0.031	0.050	0.810
γ-GT, U/l	Before	21.00 (15.00, 35.10)	23.50 (18.00, 35.10)	20.00 (16.00, 40.75)				
	After	18.00 (11.75, 25.50)	19.50 (14.00, 28.50)	18.50 (12.00, 29.25)				
	Change	−3.00 (−6.75, −0.50)	−3.00 (−12.25, −1.00)^*^	−2.50 (−9.00, 1.00)^*^	0.240	–	–	–
ALP, U/l	Before	77.50 (66.50, 94.25)	81.00 (69.50, 94.25)	70.00 (54.25, 94.25)				
	After	69.85 (60.46, 88.92)	71.87 (60.85, 89.67)	64.76 (51.73, 84.08)				
	Change	−7.15 (−15.11, −2.04)^*^	−10.32 (−17.01, −4.75)^*^	−4.15 (−11.72, 2.59)^*^	0.531	–	–	–
TBIL, μmol/l	Before	11.35 (8.35, 16.78)	12.20 (10.43, 16.78)	15.45 (10.80, 21.85)				
	After	10.35 (7.43, 12.08)	10.40 (7.53, 15.15)	12.85 (7.73, 20.20)				
	Change	−2.45 (−5.00, 0.25)^*^	−1.25 (−4.20, 0.60)	−2.05 (−7.45, 1.33)^*^	0.711	–	–	–
IBIL, μmol/l	Before	6.7 (4.48, 11.10)	7.95 (6.33, 11.10)	10.40 (6.38, 16.73)				
	After	6.90 (4.83, 9.1)	7.30 (5.40, 10.85)	9.40 (5.60, 14.70)				
	Change	−1.35 (−3.18, 2.03)	−3.00 (−2.08, 1.00)	−0.05 (−5.00, 1.63)	0.627	–	–	–
DBIL, μmol/l	Before	4.6 (4.10, 5.20)	4.35 (3.98, 5.20)	4.80 (3.83, 6.58)				
	After	3.00 (2.48, 3.60)	2.80 (2.30, 4.00)	3.05 (2.25, 4.33)				
	Change	−1.75 (−2.03, −1.18)^**^	−1.40 (−2.20, −0.45)^**^	−1.55 (−2.60, −0.95)^**^	0.393	–	–	–
TC, mmol/l	Before	4.79 (4.25, 5.39)	4.64 (4.47, 5.39)	4.39 (4.12, 4.78)				
	After	4.94 (4.31, 5.63)	4.57 (3.95, 4.92)	4.68 (4.20, 4.93)				
	Change	0.11 (−0.57, 0.51)	−0.01 (−0.43, 0.31)	0.28 (−0.22, 0.55)	0.421	–	–	–
TG, mmol/l	Before	1.84 (1.11, 2.31)	1.62 (1.03, 2.31)	1.23 (1.05, 2.27)				
	After	1.83 (1.08, 2.22)	1.56 (1.06, 2.05)	1.33 (0.98, 2.91)				
	Change	0.05 (−0.45, 0.29)	0.04 (−0.36, 0.40)	0.21 (−0.09, 0.57)^*^	0.093	–	–	–
HOMA-IR	Before	4.31 (2.90, 6.47)	3.75 (2.01, 5.45)	3.14 (1.93, 6.35)				
	After	2.97 (2.11, 4.12)	1.35 (0.49, 3.24)	0.74 (0.26, 5.78)				
	Change	−1.23 (−2.59, 0.38)^*^	−2.01 (−3.33, −0.35)^**^	−1.66 (−2.91, −0.15)^*^	0.515	–	–	–

a *Values are median (Q1–Q3) for variables with skewed distribution. TP, total protein; AST, aspartate aminotransferase; ALT, alanine aminotransferase; γ-GT, gamma-glutamyl transferase; ALP, alkaline phosphatase; TBIL, total bilirubin; IBIL, serum indirect bilirubin; DBIL, serum direct bilirubin; TC, total cholesterol; TG, triglyceride; HOMA-IR, homeostatic model assessment of insulin resistance = FINS (uU/ml) × FGlucose(mmol/l)/22.5.*

* *means comparison between before and after the intervention, P < 0.05;*

** *means comparison between before and after the intervention, P < 0.001. Paired t-test was used for comparison before and after the intervention.*

b *means the low-dose group (250 mg/day);*

c *means the medium-dose group (1,000 mg/day);*

d *means the high-dose group (2,000 mg/day); P^group^ means comparison among the three groups; P^1v2^ means comparison between the low-dose group and the medium-dose group; P^2v3^ means comparison between the medium-dose group and the high-dose group; P^1v3^ means comparison between the low-dose group and the high-dose group. Among-group differences were analyzed with ANOVA for normally distributed variables or the Wilcoxon rank-sum test for skewered variables. The Student–Newman–Keuls test method was used for post-hoc testing to complete the paired comparison between the groups*.

### Effects on Glucose Metabolism

**Figure 3:**
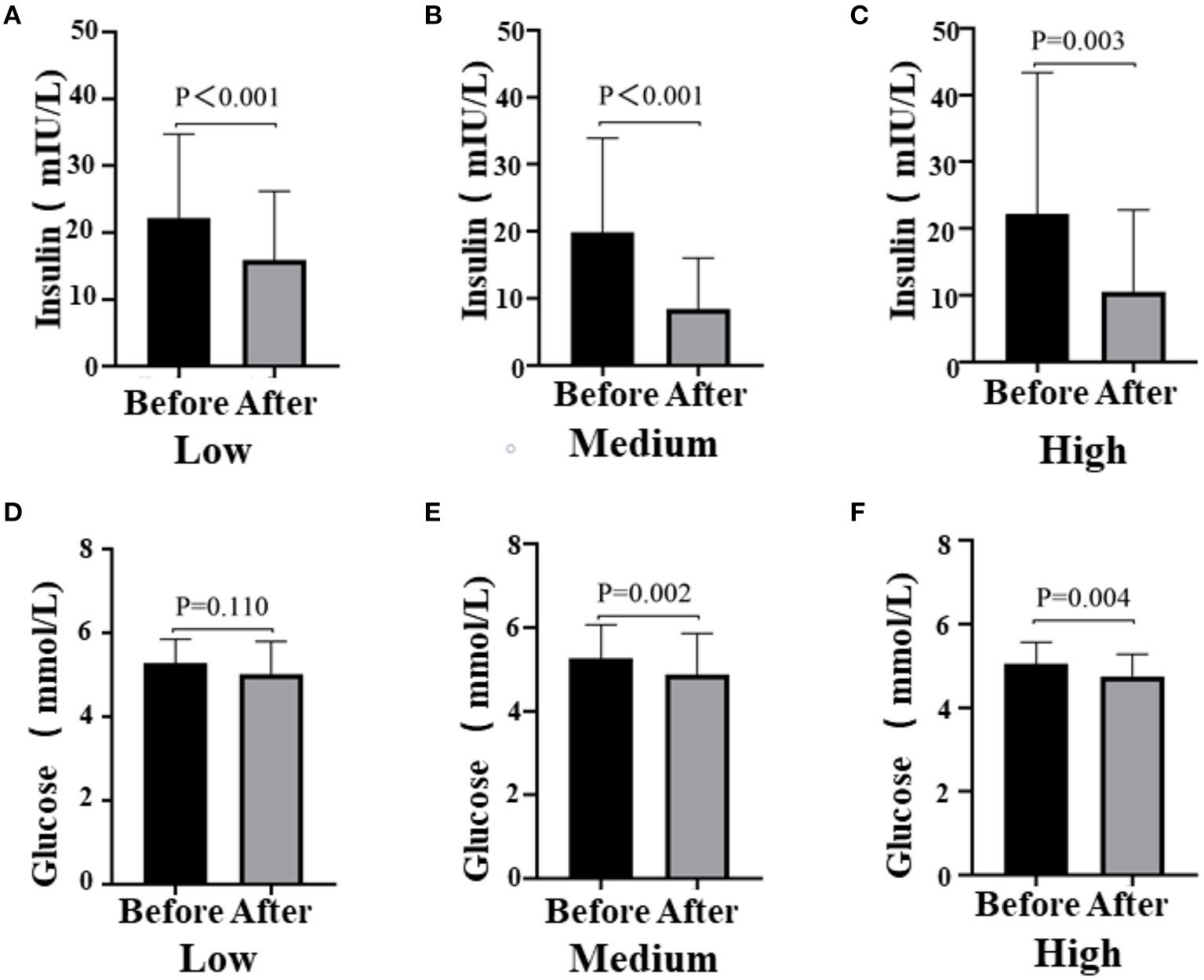
Fasting blood glucose levels (**A**, Low dose group; **B**, Medium dose group; **C**, High dose group) and insulin levels (**D**, Low dose group; **E**, Medium dose group; **F**, High dose group) before and after the intervention in all three groups, respectively. Black means glucose or insulin plasma concentration (mmol/l, mIU/l) before the intervention, gray means glucose or insulin plasma concentration after the intervention; paired *t*-test used to test the difference before and after the intervention in the three groups.

**Figure 4:**
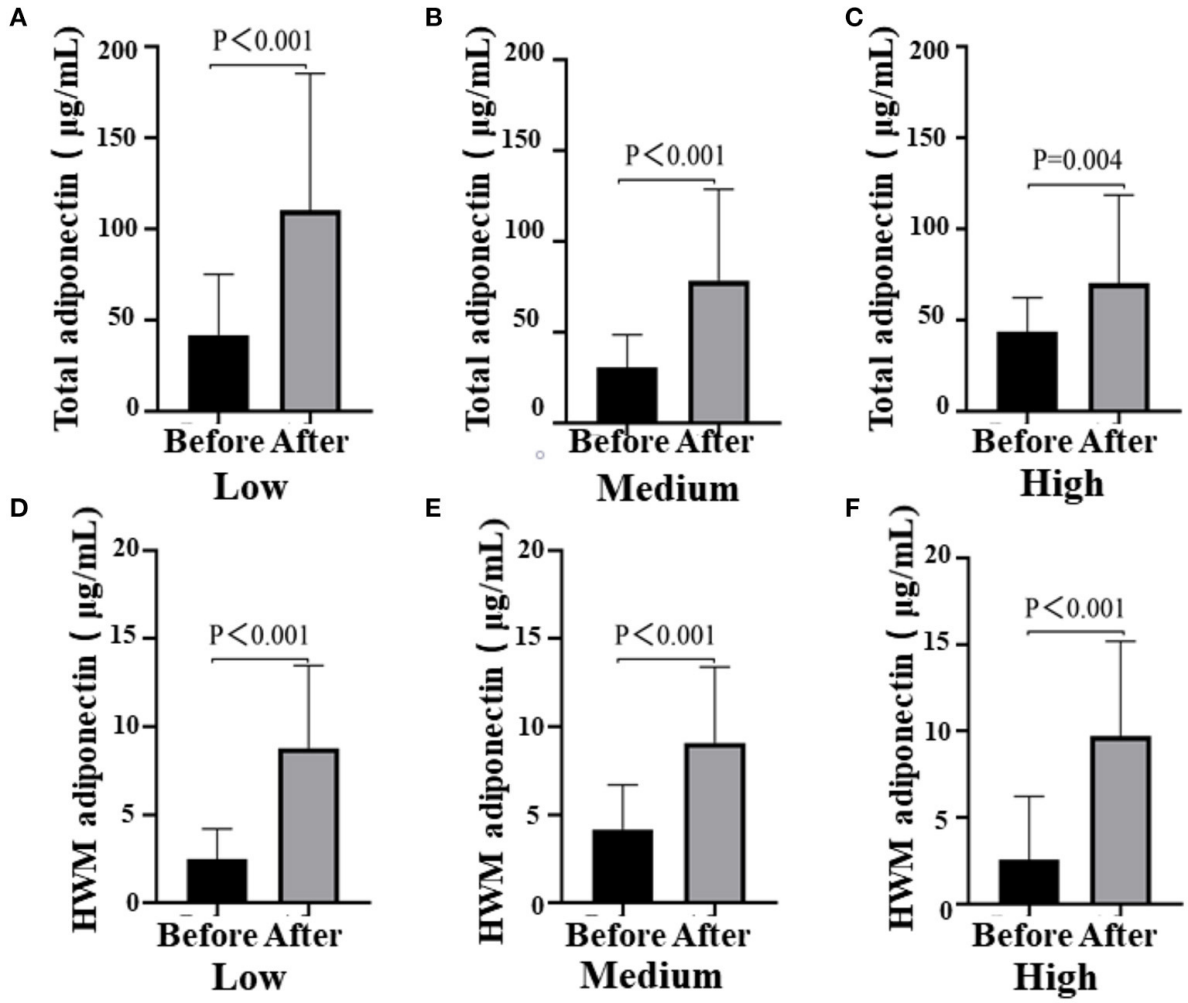
Total adiponectin (**A**, Low dose group; **B**, Medium dose group; **C**, High dose group) and HWM adiponectin levels (**D**, Low dose group; **E**, Medium dose group; **F**, High dose group) before and after the intervention in the three groups, respectively. Black means total adiponectin or HWM adiponectin plasma concentration (μg/ml) before the intervention, gray means total adiponectin or HWM adiponectin plasma after the intervention; paired *t*-test used to test the difference before and after the intervention in all three groups.

After the intervention, blood glucose metabolism status statistically significantly improved in all three groups (Figure 3). The blood glucose levels were significantly decreased in the Medium group (before 5.05 ± 0.51 vs. after 4.75 ± 0.53, *P* = 0.002) and the High group (before 5.26 ± 0.80 vs. after 4.88 ± 0.98, *P* = 0.004), while the insulin levels of participants in all the groups reduced obviously [Low (before 22.24 ± 12.50 vs. after 15.92 ± 10.27, *P* < 0.001); Medium (before 19.22 ± 14.54 vs. after 8.46 ± 7.55, *P* < 0.001); High (before 22.24 ± 21.1 vs. after 10.54 ± 12.45, *P* < 0.003)]. However, there was no difference among groups (glucose, *P* = 0.753; insulin, *P* = 0.389; data not shown). Notably, there was a remarkable decrease in homeostasis model assessment for insulin resistance (HOMA-IR) among the three groups, especially the Medium group (before 4.61 ± 3.51 vs. after 2.62 ± 2.41, *P* < 0.001; Table 2). Then, we also detected the HWM adiponectin and total adiponectin levels of patients and found a significant elevation after the intervention with VC in all groups [total adiponectin, Low (before 41.77 ± 33.26 vs. after 110.41 ± 74.87, *P* < 0.001), Medium (before 30.57 ± 17.93 vs. after 78.48 ± 50.24, *P* < 0.001), High (before 43.57 ± 18.76 vs. after 70.29 ± 48.25, *P* < 0.001); HWM adiponectin, Low (before 2.49 ± 1.73 vs. after 8.79 ± 4.69, *P* < 0.001), Medium (before 4.18 ± 2.54 vs. after 9.09 ± 4.31, *P* < 0.001), High (before 2.57 ± 3.65 vs. after 9.70 ± 5.49, *P* = 0.004)]. However, the elevation of both total and HMW adiponectin was not remarkable among the groups (data not shown) (Figure 4).

### Changes in the Intestinal Microbial Community

**Figure 5:**
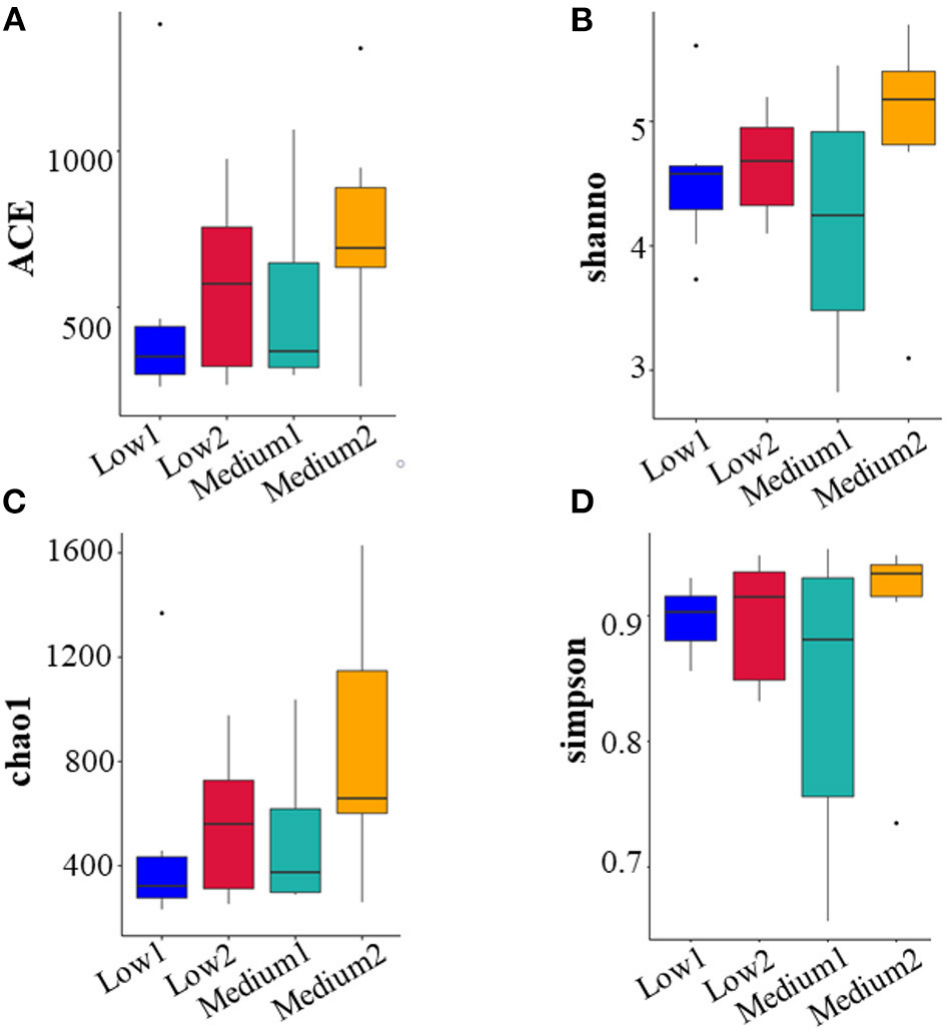
Analysis and comparison of multiple indexes before and after the intervention in the Low and the Medium groups. The study of microbial diversity in community ecology can reflect the abundance and diversity of microbial communities through a single sample diversity analysis [α(alpha) diversity], including a series of statistical analysis indexes to estimate the species abundance and diversity of environmental communities diversity. The picture showed the four indexes [ACE **(A)**, shannon **(B)**, chao1 **(C)**, and simpson **(D)**] of the α diversity analysis of 6 subjects in the Low group and Medium group respectively before and after the intervention. The higher the values of the four indexes, the more abundant species in the community. Low1 means before the intervention in the low-dose group, Low2 means after the intervention in the low-dose group, Medium1 means before the intervention in the medium-dose group, and Medium2 means after the intervention in the medium-dose group.

**Figure 6:**
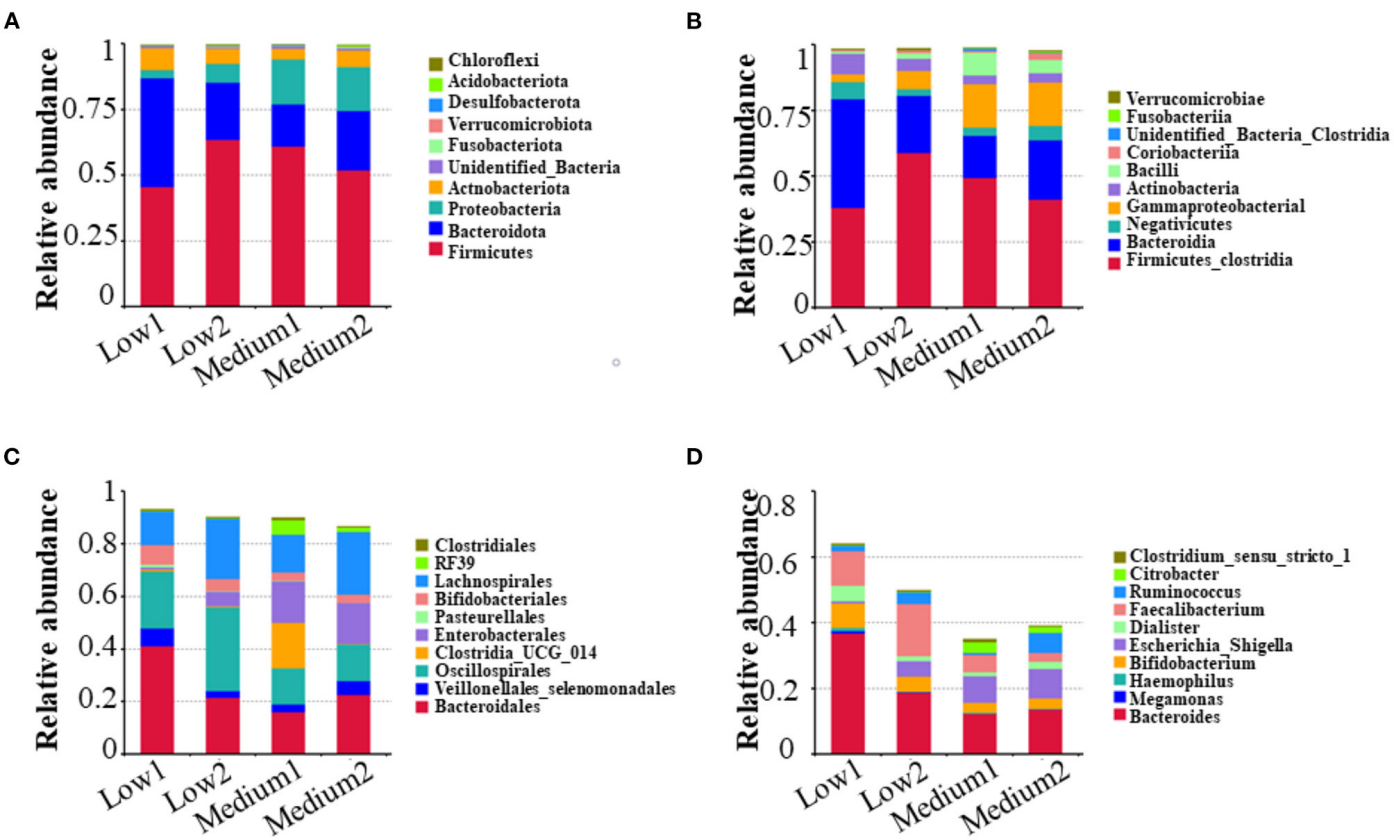
Analysis and comparison of relative abundance of top 10 before and after the intervention in the Low and the Medium groups at phylum, class, order, and genus level. The higher the relative proportion, the more abundance in the community at different biological classification level. **(A)** means phylum level; **(B)** means class level; **(C)** means order level; and **(D)** means genus level.

For the 16S rRNA sequencing of the intestinal microbiota of patients with NAFLD, there were six volunteers in the Low and the Medium groups, respectively, and 24 fecal biological samples were collected. The alpha diversity analysis indicated that all the four diversity indexes (ACE, Shannon, chao1, and Simpson) in the two groups showed an upward trend (Figure 5). For species community analysis (Figure 6), compared with the control group (250 mg/day), the Bacteroides level in the middle-dose group declined, the Firmicutes rose, and the proportion of Firmicutes and Bacteroides (Firmicutes to Bacteroidetes, F/B) decreased.

## Discussion

In this study, we observed that oral VC supplementation improved liver health and glucose metabolism in patients with NAFLD. The intake of 1,000 mg/day VC showed a profound effect. Vitamin C supplementation increased plasma VC, adiponectin, HMW adiponectin levels, and the diversity of intestinal microbiota. Furthermore, decreased the majority of parameters associated with liver function, fasting glucose level, and HOMA-IR. Among the participants, men were likely to get more benefits from the supplementation.

Regarding the nutritional risk factors, excessive energy intake but relatively minor intake of micronutrients contributes more to NAFLD (33). Hence, lifestyle changes and antioxidant therapy are widely used to prevent and treat NAFLD (34). Among antioxidants, vitamin E is now widely recommended clinically at present. The largest trial by now revealed that vitamin E treatment showed a superior effect than pioglitazone on improving histological and biochemical features in subjects with non-alcoholic steatohepatitis (NASH) (35). However, vitamin E would increase insulin resistance and plasma triacylglycerols when administered for 2 years and even was associated with excess mortality in primary and secondary prevention trials (36, 37). Compared with fat-soluble vitamin E that can accumulate after long-term use, VC is water-soluble and remarkably safe, even at 10–100 times the recommended dietary allowance when taken orally (38). Some previous studies have indicated that the combined supplementation of VC and other nutrients, such as vitamin E or resveratrol, can alleviate hepatic steatosis, but the effect of VC alone on liver function was still not apparent (39, 40). Animal experiments showed that VC was involved in regulating circulating and hepatic lipids homeostasis (41). In mice, a moderate dose (30 mg/kg/day) was beneficial for the preventing and treating of NAFLD caused by a high-fat diet (42). Although the epidemiological study also supported the association between inadequate VC intake and NAFLD, there is no direct evidence that can prove the benefits of supplementing VC on patients with NAFLD (43). In our study, we found that 12 weeks of VC intervention remarkably improved the indicators of liver function in patients with NAFLD. Our results suggested that as adjuvant therapy for daily health prevention and improvement of prognosis of NAFLD, routine supplementation of VC can help the recovery of liver function.

Non-alcoholic fatty liver disease is associated with an increased risk of cardiovascular disease, dyslipidemia, and T2DM in adults (44, 45). Several hypotheses were generating to understand the metaorganism pathways that influence the development and progression of NAFLD. What has been determined is that it is strongly associated with obesity and insulin resistance (46–48). Consistently, in this study, we also observed that all the patients were overweight, and their waist circumferences were very close to the cut-off point according to the Chinese standard. Since all participants were asked to keep their dietary habits, they had rare weight changes no matter how much VC was administered. In terms of glucose homeostasis, we found the fasting plasma insulin levels were close to the upper level of normal standard, even though their fasting glucose levels were still normal. It appeared likely that those patients had a high risk of getting the possibility to progress to T2DM soon, which was consistent with the epidemiological fact that patients with NAFLD are more likely to develop other metabolic diseases, especially T2DM (49). Interestingly, our results showed that 12 weeks of VC supplementation improved the glucose metabolism of patients. Specifically, fasting plasma insulin and glucose contents (except for the Low group) were all reduced after the intervention markedly, accompanied by a lower degree of insulin resistance. Similarly, the most favorable effect was still seen in the Medium group. Although it was hard to tell the causal relationship between the recovery of liver function and improved glucose metabolism, our results at least indicated that the intervention of VC would decelerate the disease progression of patients with NAFLD.

Previous studies suggested that adiponectin, which has many favorable effects on metabolic diseases, may be involved in the pathogenesis of NAFLD (22, 23). Our previous study showed that VC pretreatment might be a new treatment method of NAFLD/NASH through attenuate hepatocyte stress induced by TNFα via activation of the FGF21/FGFR2/adiponectin pathway (24). Expectedly, in this study, after the intervention, even at the smallest dose (250 mg/day), total adiponectin and HWM adiponectin levels showed a significant upward trend, which may contribute to the improvement of both liver function and glucose metabolism in patients with NAFLD. To the best of our knowledge, this is the first time to report the effect of VC supplementation on adiponectin production in human individuals, which is of great significance to clarify the protective role of VC in NAFLD. More interestingly, we found that the men seemed to get more benefits from VC supplementation than the women. This finding may be explained by the truth that men generally consume fewer fresh vegetables and fruits daily than the women at baseline (data not shown), and their initial VC levels were lower. Our findings were consistent with a recent study that showed that the inverse association between dietary VC intake and NAFLD in middle-aged and older adults was stronger in the male population (10).

Gut microbiota and gut-liver axis dysfunction are critical for NAFLD progression and the development of more severe inflammatory and fibrotic stages NASH (50). Several studies have suggested that in addition to changes in adiponectin, changes in the intestinal microbiota may also be the driving force for the progress of NAFLD (28, 29). Our research also found that taking 250 mg/day and 1,000 mg/day of VC daily for 12 weeks can improve the intestinal microbiota of patients with NAFLD by increasing the diversity of intestinal microbiota and the relative proportion of beneficial bacteria. Bacteroidetes and Firmicutes are the dominant “good” bacteria and “bad” bacteria, respectively. In the human intestinal tract, a person with NAFLD showed a higher Firmicutes to Bacteroidetes (F/B) ratio than a healthy population (51, 52). In this study, we found that only 1,000 mg/day of VC supplementation for 12 weeks increased the diversity of species and the dominant microbiota. Unfortunately, only 12 people from the Low and Medium groups provided stool samples. Although the sample size was small, our results hint that VC supplementation may produce beneficial effects on patients with NAFLD by affecting the intestinal microbiota, which may, in turn, promote liver function and other related metabolic parameters in patients with NAFLD through the gut-liver axis.

The main strength of our study was the randomized controlled design and stratification by age, gender, and BMI, which eliminates interindividual differences. Furthermore, our research filled the gap in the trial of VC intervention alone in patients with NAFLD and revealed the beneficial effects. However, there were also some limitations in our research. First, there was no placebo control in our study because it was challenging to make a placebo with the same odor and color as VC. Regardless, it was also appropriate to take a dose that is close to the PI-NCD of VC (200 mg/day) to meet the favorable principles in ethics. Second, due to the relatively small group of participants, the differences in couples of indicators regarding liver function were not significantly different though a good effect was present. To obtain more generalizable conclusions, more extensive population studies are still needed.

In conclusion, to our best knowledge, this is the first study that determined the effect of oral VC supplementation on patients with NAFLD. Daily supplementation with VC, especially 1,000 mg/day, can help promote liver function recovery and glucose homeostasis in patients with NAFLD.

## Data Availability Statement

The raw data supporting the conclusions of this article will be made available by the authors, without undue reservation.

## Ethics Statement

The studies involving human participants were reviewed and approved by the Ethics Committee of Xi'an Jiaotong University and Xianyang Central Hospital. The patients/participants provided their written informed consent to participate in this study.

## Author Contributions

XLu, ZH, and XLi designed the research. ZH, HY, SW, ZX, PW, XG, WZ, and JG conducted the research. ZH, XLi, and HY analyzed the data and performed the statistical analysis. XLi, ZH, and XLu wrote the paper and had primary responsibility for the final content. All the authors read and approved the final manuscript.

## Funding

This work was supported by the National Natural Science Foundation of China (Grant No. 81874263).

## Conflict of Interest

The authors declare that the research was conducted in the absence of any commercial or financial relationships that could be construed as a potential conflict of interest.

## Publisher's Note

All claims expressed in this article are solely those of the authors and do not necessarily represent those of their affiliated organizations, or those of the publisher, the editors and the reviewers. Any product that may be evaluated in this article, or claim that may be made by its manufacturer, is not guaranteed or endorsed by the publisher.

## Abbreviations

NAFLD: nonalcoholic fatty liver disease
VC: vitamin C
RCT: randomized controlled trial
HMW: high molecular weight
AST: aspartate aminotransferase
ALT: alanine aminotransferase
γ-GT: gamma-glutamyl transferase
ALP: alkaline phosphatase
TBIL: total bilirubin
DBIL: direct bilirubin
HOMA-IR: homeostasis model assessment for insulin resistance
T2DM: type 2 diabetes
PI-NCD: proposed intake for chronic non-communicable disease
UHPLC: ultrahigh performance liquid chromatography
OTUs: operational taxonomic units
TG: triglycerides
NASH: nonalcoholic steatohepatitis.
