# Supplementary material for: Effects of Oral Vitamin C Supplementation on Liver Health and Associated Parameters in Patients With Non-Alcoholic Fatty Liver Disease: A Randomized Clinical Trial
Source: Front Nutr. 2021 Sep 14;8:745609. doi: 10.3389/fnut.2021.745609 (PMC8478121; doi:10.3389/fnut.2021.745609)
Supplement: Supplementary file 1 [file Data_Sheet_1.DOCX]

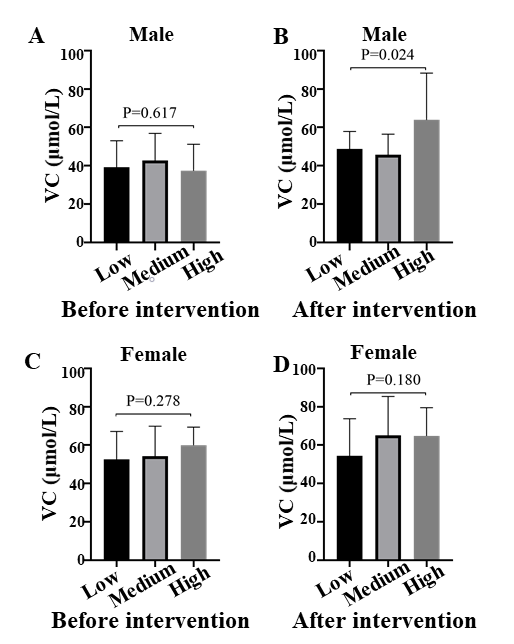


**SUPLLYMENTARY FIGURE 1** Plasma VC levels of different gender among three group before and after intervention.

The one-way analysis of variance and Student-Newman-Keuls (SNK-q) test were used to determine the significance of the difference among three groups; Abbreviation: VC, Vitamin C
